# Smartphone-Based Assessment of the Stretch–Shortening Cycle: Validity and Reliability of the My Jump Lab App for Measuring the Dynamic Rebound Index

**DOI:** 10.3390/s26103068

**Published:** 2026-05-12

**Authors:** Carlos Balsalobre-Fernández

**Affiliations:** Applied Biomechanics and Sport Technology Research Group, Autonomous University of Madrid, 28049 Madrid, Spain; carlos.balsalobre@uam.es

**Keywords:** stretch-shortening cycle, smartphone, plyometrics, monitoring

## Abstract

**Highlights:**

**What are the main findings?**

- The My Jump Lab smartphone application showed very large to nearly perfect validity for measuring jump height, contact time and Dynamic Rebound Index (DRI) during drop jumps compared to a force platform.
- The app demonstrated good to high reliability, both between sessions and within-session attempts, with low measurement error.

**What are the implications of the main findings?**

- My Jump Lab can be used as a practical and cost-effective alternative to force platforms for assessing drop jump performance and DRI in applied settings.
- The accessibility of smartphone-based assessment may facilitate wider monitoring of stretch-shortening cycle performance in sport and research contexts.

**Abstract:**

The aim of the present study was to analyze the validity and reliability of the My Jump Lab (My Jump Lab, Madrid, Spain) smartphone application for measuring the Dynamic Rebound Index (DRI) during drop jump testing. Seventeen physically active participants completed two testing sessions separated by 48 h. In each session, six drop jumps from a 40 cm box were performed while jump height, contact time and DRI were simultaneously recorded using a force platform and the app. Very large to nearly perfect correlations were observed between devices for all variables (r > 0.98). Agreement between methods was excellent, as indicated by the intraclass correlation coefficient (ICC > 0.97) and the concordance correlation coefficient (CCC > 0.93). Bland–Altman analysis revealed small systematic differences and narrow limits of agreement. The root mean square error (RMSE) was low, indicating minimal prediction error. Test–retest reliability between sessions was good for both devices (ICC = 0.825–0.925), and within-session reliability across attempts was high (ICC = 0.705–0.870). These findings indicate that My Jump Lab provides valid and reliable measurements of drop jump performance, including DRI, relative to a force platform, with potential utility in applied settings.

## 1. Introduction

Drop jump (DJ) tests are widely used to assess stretch–shortening cycle (SSC) function and reactive strength in athletic populations [1,2,3]. During the DJ, athletes are required to rapidly transition from an eccentric to a concentric muscle action after landing from a predetermined height. This ability has been associated with performance in sprinting, change in direction and jumping tasks, making the DJ one of the most commonly used assessments in strength and conditioning settings [3,4,5,6].

Traditionally, reactive strength during the DJ has been quantified using the reactive strength index (RSI), calculated as the ratio between jump height and contact time. The RSI is a simple and widely adopted metric that has been shown to be sensitive to training adaptations and fatigue [7,8,9,10]. However, several limitations have been highlighted [11]. First, RSI does not account for drop height, making comparisons between tests performed from different box heights difficult. Second, RSI can be artificially increased by reducing contact time without a meaningful improvement in jump height, which may lead to misinterpretations of SSC performance.

To overcome these limitations, new metrics have recently been proposed to provide a more comprehensive assessment of DJ performance. Among them, the Dynamic Rebound Index (DRI) has gained attention as a novel indicator that integrates jump height, contact time and drop height into a single outcome [11]. This approach allows for a more standardized evaluation of SSC function across different testing conditions and may provide a more accurate representation of an athlete’s reactive capabilities. Despite its potential relevance, the measurement of DRI has been limited to force platforms, which are generally considered the gold standard for biomechanical assessment but are often expensive and not easily accessible in applied environments [1,12]. However, a potential limitation of the DRI is that it incorporates drop height in its calculation, which may introduce additional sources of error if this variable is not accurately measured. While force platforms or motion capture systems allow precise estimation of the actual drop height, in applied settings practitioners often assume a fixed value based on box height. This simplification may lead to discrepancies between the theoretical and actual drop height due to individual landing strategies and movement patterns. Furthermore, although the DRI has been proposed as an alternative to the reactive strength index, the relationship between both metrics remains unclear. Given that RSI is widely used in both research and practice, understanding how DRI relates to RSI may provide additional context for the interpretation and practical adoption of this novel metric.

In recent years, smartphone applications have emerged as practical and affordable alternatives for field-based performance testing. Previous research has demonstrated that mobile apps can provide valid and reliable measurements of jump height and other performance variables when compared with force platforms [13,14,15,16]. Specifically, the My Jump Lab application has been extensively validated versus reference devices (such as force platforms) for the assessment of vertical jump performance using video-based analysis [17,18,19,20,21,22] with demonstrated validity for drop jump testing, including the measurement of contact time, jump height, and the reactive strength index. However, to the best of our knowledge, no study has examined the validity and reliability of a smartphone application for the measurement of DJ-specific variables such as contact time or derived indices such as the DRI.

Therefore, the aim of the present study was to analyze the validity and reliability of the My Jump Lab smartphone application for the measurement of jump height, contact time and the DRI during drop jump testing, using a force platform as the criterion method. In addition, the influence of using actual versus box-derived drop height on DRI calculation was examined, and the relationship between DRI and the traditional reactive strength index was explored. We hypothesized that the My Jump Lab application would be highly valid and reliable for measuring jump height, contact time, and the DRI.

## 2. Materials and Methods

### 2.1. Design

This study employed a repeated-measures design to analyze the validity and reliability of a smartphone application for the assessment of drop jump performance. Participants attended two testing sessions separated by 48 h. In each session, six drop jumps were performed while jump height, contact time and the DRI were simultaneously recorded using a force platform and the My Jump Lab app (My Jump Lab, Madrid, Spain). A total of 204 drop jumps were analyzed for validity purposes. Additionally, test–retest reliability between sessions and within-session reliability across attempts were evaluated for both devices.

### 2.2. Participants

Seventeen physically active participants (age = 22.5 ± 1.2 years; height = 1.77 ± 0.07 m; body mass = 72.3 ± 7.5 kg) volunteered to participate in this study. All participants were familiar with drop jump testing and had previous experience with plyometric exercises. Participants were recreationally active sports science students who regularly engaged in resistance training and jumping-based activities as part of their academic and recreational practice. Participants were instructed to avoid strenuous physical activity in the 24 h prior to each testing session.

### 2.3. Sample Size Estimation and Justification

In this study, we did not perform a formal a priori sample size calculation. Instead, we determined the sample size pragmatically based on feasibility and in reference to previous validation studies of the My Jump/My Jump Lab applications. These studies typically included samples of 10 to 20 participants and approximately 100 to 120 total observations. Notably, these studies reported very high agreement with laboratory-grade equipment [14,23,24]. Based on this precedent, 17 physically active participants were recruited. The study design also planned the number of repeated observations a priori. Each participant performed six drop jumps in two testing sessions, separated by 48 h, resulting in 204 paired measurements. This approach was chosen to provide a sufficient number of paired observations for method-comparison analyses while also allowing for the assessment of within-session and between-session reliability. Although no formal a priori power analysis was conducted, the sample size and repeated-measures structure were deemed appropriate for the study’s objectives and aligned with previous validation research in this field.

### 2.4. Procedures

Participants attended two testing sessions separated by 48 h, performed at the same time of day to minimize potential circadian effects. Each session began with a standardized warm-up consisting of dynamic stretches and low-intensity lower-body exercises. Following the warm-up, participants performed six drop jumps from a 40 cm box. For each jump, participants were instructed to step forward from the box, land with both feet simultaneously, and immediately perform a maximal vertical jump while minimizing ground contact time. Hands were placed on the hips throughout the entire movement to eliminate arm swing. A rest period of approximately 10 s was allowed between repetitions to minimize fatigue effects. All jumps were simultaneously recorded using a force platform and the My Jump Lab app. Trials were deemed valid when participants stepped out of the box without excessive prior counter-movement and completed the jump with fully extended knees during the flight phase, maintaining balance and avoiding visible technical errors.

### 2.5. Instruments and Data Processing

A dual force platform system (Hawkin Dynamics, Westbrook, ME, USA) sampling ground reaction forces at 1000 Hz was used as the criterion method in the present study. Force-time data were collected and processed using the manufacturer’s proprietary software. Jump height and contact time were calculated from the vertical ground reaction force (GRF) signal using standard procedures. The DRI was subsequently derived from these variables as described by Brooks [11]:

$$DRI=\frac{h+h_{drop}}{g{tc}^{2}}$$

where *h* is the jump height, *h*_drop_ is the drop height (derived from the force platform by calculating the center of mass velocity at ground contact and fixed at 0.40 m—i.e., box height—when using the app), *g* is gravitational acceleration, and *tc* is the contact time.

**Figure 1:**
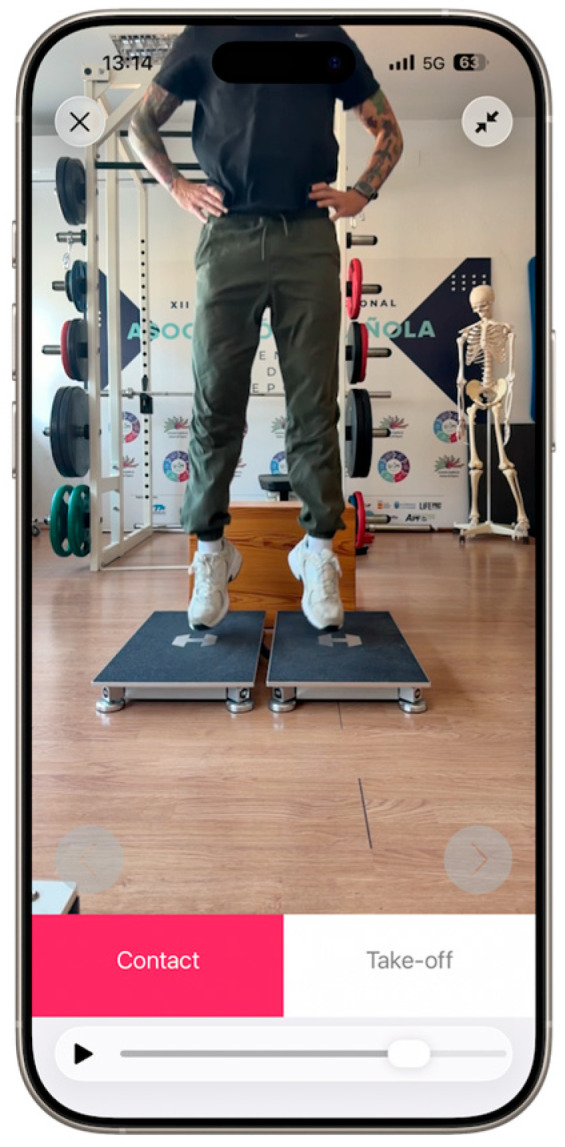
User interface of the My Jump Lab application, displaying the fundamental setup of the instruments utilized in the current investigation. This includes the placement of the force platform and the positioning and placement of the camera.

The My Jump Lab app v 5.0.6 (My Jump Lab, Madrid, Spain) was installed on an iPhone 17 Pro Max running iOS 26.3 (Apple Inc., Cupertino, CA, USA). The device was mounted on a tripod and positioned to record the frontal plane of the participants. The camera was positioned 0.2 m above the floor and 1.5 m away from the participant, ensuring that the entire jump was captured within its field of view. The app measures jump performance by recording video at a high frame rate of 240 frames per second with a resolution of 1920 × 1080. Then, it manually inspects the take-off and landing frames to determine the exact moments of take-off and landing. Take-off was defined as the first frame in which both feet were in the air, while contact was defined as the first frame in which at least one foot made contact with the ground. Using these instants, the app calculates the jump height and contact time. See Figure 1 for details. This procedure has been extensively validated in the literature to assess the performance of various types of jumps, including the DJ [25]. The DRI was automatically computed by the app using the corresponding jump height, contact time and box height values. All data from both devices were exported to CSV files and organized for subsequent statistical analysis.

### 2.6. Statistical Analyses

All data are presented as mean ± standard deviation (SD). Concurrent validity between the My Jump Lab app and the force platform was assessed using Pearson’s correlation coefficients (r) and two-way mixed-effects, single-measurement intraclass correlation coefficients (ICC 3,1). In addition, Lin’s concordance correlation coefficient (CCC) was calculated to assess agreement between methods. Linear regression analysis was performed to derive an equation to estimate force platform values from app measurements, and the root mean square error (RMSE) was calculated as an indicator of prediction error. Systematic bias and agreement between methods were further evaluated using Bland–Altman analysis, which involved calculating mean differences (bias) and limits of agreement (LoA). Additionally, test–retest reliability between sessions was assessed using two-way mixed-effects, average-measurement intraclass correlation coefficients (ICC 3,k), and coefficient of variation (CV). Within-session reliability across the six attempts was evaluated using ICC (3,1) and CV. Statistical analyses were performed using JASP 0.96 for Apple Silicon (University of Amsterdam, Amsterdam, The Netherlands). A large language model (ChatGPT 5.2, OpenAI, San Francisco, CA, USA) was used to assist with language editing during the preparation of this manuscript. It was not used for data analysis or statistical procedures.

## 3. Results

Within-session reliability results for both devices are presented in Table 1. Overall, reliability across the six attempts was high for jump height and DRI in both the force platform and My Jump Lab, with ICC values ranging from 0.810 to 0.870. Contact time showed lower reliability, with ICC values of 0.705 for both devices. The CV ranged from 5.8% to 7.0% for jump height and contact time and was higher for DRI (11.5–12.8%).

Concurrent validity results are shown in Table 2. Very large to nearly perfect correlations were observed between the My Jump Lab app and the force platform for all variables (r = 0.979–0.994). Agreement between methods was excellent, as indicated by the intraclass correlation coefficient (ICC = 0.974–0.994) and the concordance correlation coefficient (CCC = 0.932–0.978).

**Figure 2:**
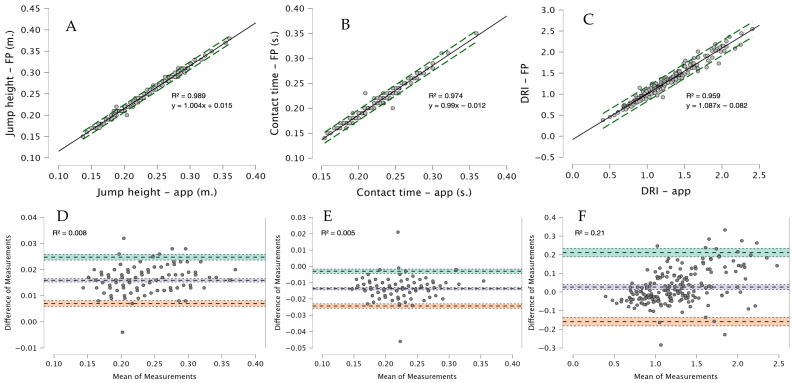
Correlation and agreement between My Jump Lab and force platform measurements for jump height, contact time, and DRI. Panels (**A**–**C**) show scatter plots with R^2^ values, regression equations, and 95% prediction intervals (green dashed lines). Panels (**D**–**F**) show Bland–Altman analyses with R^2^ values for heteroscedasticity assessment and 95% confidence intervals for the bias and limits of agreement (shaded areas).

Small systematic differences were observed between methods. My Jump Lab slightly underestimated jump height (bias = −0.016 m) and DRI (bias = −0.026), while contact time was slightly overestimated (bias = 0.014 s). The RMSE was low across all variables (0.005–0.08), indicating minimal prediction error. Scatter plots with regression lines for all variables are presented in Figure 2. Bland–Altman analysis confirmed these findings, showing small biases and relatively narrow limits of agreement for all variables. For jump height and contact time, limits of agreement were tight, whereas DRI showed wider limits of agreement, reflecting greater variability in this derived metric. No clear proportional bias was observed across the range of values, as indicated by the Bland–Altman regression analyses, with very low R^2^ values (0.001–0.21), suggesting a negligible relationship between the magnitude of the measurements and the differences between methods.

Test–retest reliability results between sessions are presented in Table 3. Reliability was generally high for both devices, with ICC values ranging from 0.825 to 0.925 for jump height, 0.869 to 0.889 for contact time, and 0.847 to 0.901 for DRI. The coefficient of variation ranged from 5.8% to 10.4% for jump height and contact time, and from 12.8% to 16.1% for DRI, indicating greater variability for this variable across sessions.

Finally, a very strong association was observed between RSI and DRI for both measurement methods (force platform: r = 0.969, *p* < 0.001; My Jump Lab: r = 0.960, *p* < 0.001).

## 4. Discussion

The main findings of the present study indicate that the My Jump Lab application provides valid and reliable measurements of drop jump performance, including jump height, contact time, and the DRI, when compared with a force platform. Very high correlations, excellent ICC values, and strong agreement metrics (CCC) were observed across all variables, together with low RMSE values, supporting the accuracy of the app for field-based assessment. These findings align with previous validation studies conducted on the My Jump ecosystem, which originated from the original My Jump application [14]. These studies have since tested newer versions, including My Jump 2 and the current version, My Jump Lab. All these versions have demonstrated high validity and reliability in various studies when compared to force platforms [14,18,25,26,27]. More recent studies have extended these findings to additional variables such as interlimb asymmetry, the RSI or vertical stiffness during drop jump testing [19,25,28,29], confirming that video-based approaches can accurately capture complex neuromuscular performance metrics when properly implemented. Unlike countermovement jumps, the DJ requires precise detection of very short contact times, increasing the potential for measurement error. Despite this, My Jump Lab showed excellent agreement with the force platform for contact time (r = 0.987; ICC = 0.987), indicating that high-speed video analysis can accurately identify key temporal events even in fast SSC actions.

With respect to systematic bias, the app slightly underestimated jump height and DRI, while slightly overestimating contact time. These small differences are consistent with previous research using video-based methods, where minor discrepancies can arise from frame selection during take-off and landing detection. For jump height and contact time, the magnitude of these differences was trivial from a practical perspective, as reflected by the low RMSE values and the narrow limits of agreement observed in the Bland–Altman analysis.

In contrast, DRI showed slightly larger differences between methods, as indicated by wider limits of agreement and greater variability. This is likely explained by the nature of DRI as a composite variable, as well as by the incorporation of drop height into its calculation. One of the main contributions of this study is the validation of the DRI, a recently proposed metric designed to overcome some of the limitations of the traditional RSI. The RSI has been widely used in both research and applied settings, but it does not account for drop height and can be artificially influenced by reductions in contact time. In contrast, the DRI integrates jump height, contact time, and drop height into a single outcome, allowing for more standardized comparisons across testing conditions. As originally proposed by Brooks [11], this metric may provide a more comprehensive representation of SSC performance. The incorporation of drop height into the DRI calculation represents both a strength and a potential limitation. In the present study, the actual drop height measured using the force platform was on average 7.7 ± 2.0 cm lower than the nominal box height. This discrepancy likely contributed to the greater bias and wider limits of agreement observed for DRI, as the app assumes a fixed drop height based on box dimensions. These findings highlight that assuming a fixed drop height may introduce systematic error, likely due to individual differences in stepping strategy and center of mass displacement prior to ground contact.

Despite this discrepancy, the practical impact of this source of error appears to be limited. The correlation between DRI calculated using the actual drop height and a fixed box height was nearly perfect (r = 0.991). The mean bias between both approaches was small (−0.026), indicating that relative performance ranking between individuals is largely preserved.

Therefore, although the use of actual drop height may improve precision, the use of box height as a proxy may still be acceptable in applied settings where simplicity and feasibility are prioritized. The relationship between DRI and RSI was also examined to provide additional context for the interpretation of this novel metric. A very strong correlation was observed between both indices (r = 0.960–0.969), suggesting that they capture largely overlapping aspects of SSC performance. This is not surprising, as both metrics are derived from jump height and contact time. However, the inclusion of drop height in the DRI may provide additional information that is not captured by RSI alone, particularly when comparing performances across different drop heights. In this context, DRI may be considered an extension rather than a replacement of RSI, offering a more standardized approach to the assessment of reactive strength.

In the present study, DRI showed slightly greater variability than jump height and contact time, as reflected by higher CV values and wider limits of agreement. This finding is expected, given that DRI is a derived variable that combines multiple inputs, each with its own measurement error. Similar patterns have been reported in previous studies analyzing composite performance metrics, where error propagation leads to increased variability compared with primary variables [30,31]. Nevertheless, the agreement between methods remained very high (CCC = 0.978; ICC = 0.974), supporting the use of My Jump Lab for DRI assessment in applied settings. From a reliability perspective, both devices showed acceptable within-session and test–retest reliability, although DRI again exhibited higher variability compared with jump height and contact time. These comparatively lower ICC values might be related to the drop jump performance itself and did not seem to be influenced by the instrument used, as both the force platform and the app displayed similar within-session variability levels.

These results align with previous literature indicating that derived SSC indices tend to be more sensitive to biological and measurement variability [31,32]. Importantly, reliability values were comparable between the force platform and the app, suggesting that My Jump Lab does not introduce additional noise beyond that inherent to the measurement itself. Several limitations should be acknowledged though. First, the sample consisted of physically active young adults, which may limit generalization to elite athletes or clinical populations. Second, all testing was conducted using a single smartphone model and operating system version. Finally, although the study included repeated measurements across two sessions, no formal a priori sample size calculation was performed.

From a practical standpoint, the present findings support the use of My Jump Lab as a valid and reliable alternative to force platforms for the assessment of drop jump performance. Given the high cost and limited accessibility of force platforms, smartphone-based solutions may facilitate the widespread monitoring of SSC function in both research and applied environments. Furthermore, the ability to accurately measure DRI using a mobile app opens new possibilities for practitioners interested in more advanced and standardized metrics of reactive strength. Taken together, these findings suggest that while DRI introduces additional complexity compared to RSI, it maintains strong agreement with established metrics while offering improved standardization across testing conditions. This balance between innovation and continuity may facilitate its adoption in both research and applied settings.

## 5. Conclusions

In conclusion, My Jump Lab demonstrated high validity and reliability for the assessment of jump height, contact time, and DRI during drop jump testing when compared with a force platform. Although DRI showed slightly greater variability than primary variables, agreement between methods remained very high. These findings support the use of smartphone-based approaches for the practical monitoring of stretch–shortening cycle performance in applied settings.

## Figures and Tables

**Table 1:** Within-session reliability data using the coefficient of variation (CV) and intraclass correlation coefficient (ICC 3,1), with 95% confidence intervals (CI).

	Force Platform		My Jump Lab	
	CV (95% CI)	ICC (95% CI)	CV (95% CI)	ICC (95% CI)
Jump height (m)	6.5 (4.8–8.3)	0.870 (0.840–0.896)	6.9 (5.0–8.7)	0.851 (0.817–0.881)
Contact time (s)	7.0 (5.2–8.9)	0.705 (0.646–0.759)	5.8 (4.3–7.4)	0.705 (0.648–0.761)
DRI	12.8 (9.3–16.2)	0.817 (0.776–0.852)	11.5 (8.5–14.6)	0.810 (0.768–0.847)

**Table 2:** Concurrent validity of My Jump Lab compared with a force platform for jump height, contact time, and Dynamic Rebound Index (DRI) across 204 paired jumps.

	Force Platform (Mean ± SD)	My Jump Lab (Mean ± SD)	Bias (LoA)	RMSE	Pearson r	CCC (95% CI)	ICC (95% CI)
Jump height (m)	0.244 ± 0.043	0.228 ± 0.042	−0.016 (−0.007, −0.025)	0.005	0.994	0.932 (0.911, 0.949)	0.994 (0.992, 0.996)
Contact time (s)	0.208 ± 0.034	0.221 ± 0.033	0.014 (0.003, 0.024)	0.006	0.987	0.948 (0.930, 0.962)	0.987 (0.983, 0.990)
DRI	1.267 ± 0.434	1.241 ± 0.391	−0.026 (−0.211, 0.159)	0.08	0.979	0.978 (0.971, 0.984)	0.974 (0.965, 0.980)

Bias represents the mean difference between methods (My Jump Lab − Force Platform). Limits of agreement (LoA) were calculated as bias ± 1.96 SD of the differences according to the Bland–Altman method. r = Pearson correlation coefficient. CCC = Lin’s concordance correlation coefficient. ICC = intraclass correlation coefficient using a two-way mixed-effects model with absolute agreement (ICC (3,1)). RMSE = root mean square error. DRI = Dynamic Rebound Index. Analyses were performed using 204 paired jumps measured simultaneously with a force platform and the My Jump Lab application.

**Table 3:** Test–retest reliability between sessions, using the coefficient of variation (CV) and intraclass correlation coefficient (ICC 3,k), with 95% confidence intervals (CI). Values correspond to the mean of six drop jumps per session.

	Device	Day 1 (Mean ± SD)	Day 2 (Mean ± SD)	ICC (95%CI)	CV (95%CI)
Jump height (m)	Force platform	0.236 ± 0.046	0.260 ± 0.038	0.925 (0.756–0.977)	8.0 (5.8–10.2)
	My Jump Lab	0.220 ± 0.045	0.239 ± 0.036	0.825 (0.426–0.947)	10.4 (7.6–13.2)
Contact time (s)	Force platform	0.215 ± 0.024	0.205 ± 0.028	0.889 (0.635–0.966)	5.8 (4.3–7.3)
	My Jump Lab	0.229 ± 0.024	0.211 ± 0.029	0.869 (0.569–0.960)	7.2 (5.3–9.1)
DRI	Force platform	1.136 ± 0.305	1.343 ± 0.451	0.901 (0.677–0.970)	12.8 (9.4–16.3)
	My Jump Lab	1.112 ± 0.261	1.407 ± 0.432	0.847 (0.499–0.953)	16.1 (11.8–20.4)

## Data Availability

Raw data is available with the following Document Object Identifier (DOI): https://doi.org/10.6084/m9.figshare.31833967.

## Abbreviations

The following abbreviations are used in this manuscript:

SSC	Stretch-shortening cycle
RSI	Reactive strength index
DRI	Dynamic rebound index
GRF	Ground reaction force
SD	Standard deviation
CCC	Lin’s concordance correlation coefficient
ICC	Intraclass correlation coefficient
RMSE	Root mean square error
LOA	Limits of agreement
CV	Coefficient of variation
CI	Confidence interval
